# An Uncommon Presentation of Hamman's Syndrome in an Adolescent With Acute Diabetic Ketoacidosis and Newly Diagnosed Type 1 Diabetes

**DOI:** 10.1155/2024/1168472

**Published:** 2024-10-24

**Authors:** Daliya George, Malini Rajandran, Habib Bhurawala, Gary M. Leong

**Affiliations:** ^1^Department of Pediatrics, Nepean Hospital, Penrith, New South Wales, Australia; ^2^Discipline of Pediatrics, The University of Sydney Nepean Medical School, University of Sydney, Sydney, Australia; ^3^Faculty of Medicine, University of Notre Dame Australia, Sydney, New South Wales, Australia

## Abstract

Hamman's syndrome, a rare complication of diabetic ketoacidosis (DKA), is characterized by subcutaneous emphysema and spontaneous pneumomediastinum. This case report discusses the occurrence of Hamman's syndrome in an 11-year-old adolescent male newly diagnosed with type 1 diabetes mellitus (T1DM) and presenting with severe DKA. The patient exhibited symptoms typical of DKA, including polydipsia, polyuria, abdominal pain, and fatigue, alongside signs such as dehydration, Kussmaul breathing, and tachycardia. Following initial management with intravenous fluids and insulin infusion, he was transferred to a tertiary children's hospital for further care. Subsequently, on routine examination, he exhibited bilateral neck crepitus and a mediastinal crunching sound on auscultation, indicative of Hamman's syndrome. Conservative management led to symptom resolution, and the patient was discharged with follow-up arranged. This case highlights the importance of recognizing Hamman's syndrome as a potential complication of DKA in pediatric patients. Prompt diagnosis and management, along with differentiation from more severe conditions like Boerhaave's syndrome, are crucial for ensuring favorable outcomes. Further awareness and understanding of this rare syndrome are essential for optimal patient care and management.

**Summary**

• Hamman's syndrome is an unusual complication of DKA where presentation usually encompasses clinical features of chest or neck pain, shortness of breath, and “Hamman's sign,” a precordial crunching or popping sound during systole.
• Hamman's syndrome is a benign condition with an excellent prognosis, self-resolving with ketoacidosis correction.
• Boerhaave's syndrome should be considered as an important differential diagnosis for Hamman's syndrome, especially in cases with severe vomiting due to its high mortality rates.

## 1. Introduction

Hamman's syndrome, first described by Dr. Louis Hamman in 1939, is an uncommon yet significant complication of diabetic ketoacidosis (DKA), primarily characterized by subcutaneous emphysema and spontaneous pneumomediastinum. This syndrome typically manifests in adolescents and young adults presenting with severe DKA. Despite its rarity, Hamman's syndrome warrants attention due to its potential for serious complications and the importance of distinguishing it from other life-threatening conditions, such as Boerhaave's syndrome. This report presents a case of Hamman's syndrome in an adolescent with newly diagnosed type 1 diabetes mellitus. It underscores the critical need for heightened awareness and prompt recognition of this condition in pediatric patients presenting with DKA.

## 2. Case Presentation

An 11-year-old adolescent male presented to the emergency department (ED) in a peripheral hospital in New South Wales for assessment of sudden onset of breathlessness, throat tightness, abdominal pain, and vomiting. The adolescent reported a 2-week history of polydipsia, polyuria, abdominal pain, and tiredness. His weight was 35.7 kg (37^th^ centile), height was 147 cm (56^th^ centile), and BMI was 16.7 kg/m^2^. On examination, he was clinically dehydrated (10%), had Kussmaul breathing with tachypnea and tachycardia, and was hypertensive with an oxygen saturation of 96% on room air. His finger-prick blood sugar level (BSL) was incalculably high, and he had blood ketones of 6.0 mmol/L. A venous blood gas confirmed the provisional diagnosis of severe DKA. Blood urea and serum creatinine were elevated, as shown in Table 1.

Intravenous normal saline bolus at 20 mL/kg was administered, and insulin infusion was commenced at 0.1 unit/kg/h. In view of throat tightness being a presenting symptom, the possibility of anaphylaxis was considered, and IM adrenaline diluted to 1 : 1000 was administered as a stat dose. In view of his critical state, a decision was made to transfer his care to a tertiary children's hospital, and he was retrieved via the neonatal and pediatric emergency transport service (NETS) and admitted to the pediatric intensive care unit (PICU). Noting improvements in glucose levels, he was transitioned to subcutaneous insulin (at a rate of 1 U/kg/day) within 12 h, and subsequently, his care was transferred to the nearest local metropolitan hospital within 30 h of admission for step-down care.

**Figure 1:**
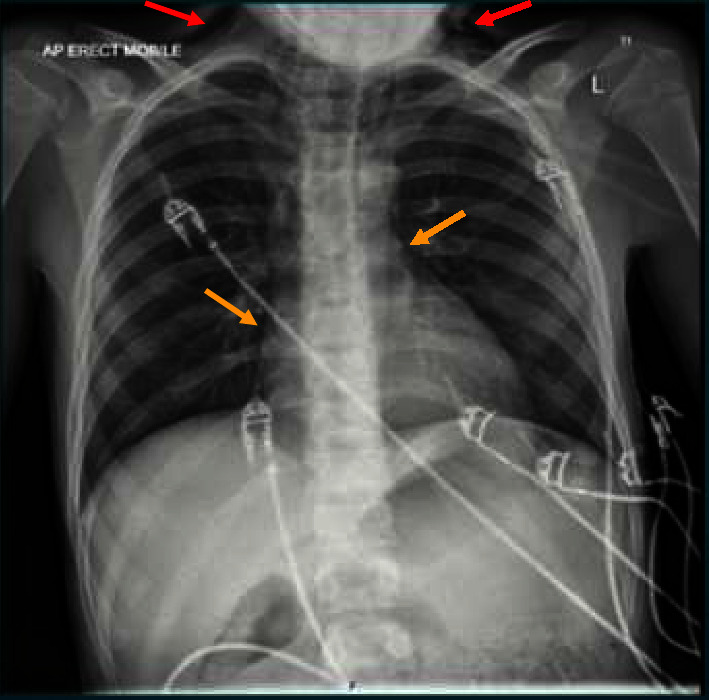
Chest X-ray AP view; red arrows point to subcutaneous emphysema in the neck and yellow arrows point to pneumomediastinum.

While in the wards, on routine rounds, an additional history of mild inspiratory chest discomfort was reported. His vital signs were normal while on room air. Clinical examination revealed bilateral neck crepitus and precordial “crunching” sound on auscultation. ECG demonstrated normal sinus rhythm. His initial chest X-ray performed when he first presented at the peripheral hospital was reviewed. This chest X-ray revealed subcutaneous emphysema of the neck and pneumomediastinum (Figure 1). He was clinically well and, hence, was conservatively managed with vigilant observation for the next 24 hours. He reported resolution of symptoms and was discharged home 72 h after diagnosis and advised follow-up with the local diabetes service.

## 3. Discussion

Hamman's syndrome is an unusual complication of DKA with constellations of subcutaneous emphysema and spontaneous pneumomediastinum that was first described by Dr. Louis Hamman in 1939 [1]. Its incidence rate is 1 : 44,000, with approximately 1 : 25,000 patients being between 5 and 34 years old with 70% of these cases occurring in males [2]. The most common physical sign of Hamman's syndrome is the “Hamman sign,” which is a mediastinal crunching sound heard during auscultation in the setting of mediastinal emphysema. Subcutaneous emphysema findings on a chest radiograph can be subtle and easily missed. Pathogenesis of Hamman's syndrome, specifically within DKA can be multifactorial. One hypothesis suggests that the higher intrathoracic pressure associated with Kussmaul breathing in DKA leads to alveoli distension and rupture, resulting in an air leak that tracks into the mediastinum [1]. A second hypothesis proposes that with severe vomiting due to ketoacidosis, the relative increase in intrathoracic pressure can cause air to track into subcutaneous tissues, causing subcutaneous emphysema [3]. A systematic review of the literature by Pauw et al. reported at least 56 published cases of spontaneous pneumomediastinum in the context of DKA which were all successfully managed conservatively, which indicates an excellent prognostic indicator for this syndrome [4]. Kamei et al. reported three similar cases in a young male with Hamman's syndrome presenting with T1DM accompanied by DKA [5]. However, it is crucial to consider Boerhaave's syndrome which is pneumomediastinum caused by an effort rupture of the esophagus as an important differential diagnosis to Hamman's, as this is a more fatal condition with a 40% mortality rate, especially when the diagnosis is delayed [6]. The management strategy for Hamman's syndrome is typically supportive, with the syndrome improving within 2–7 days without significant risk of recurrence [7]. Complications, while rare, can include a pneumothorax [8], though routine follow-up or repeat imaging is unnecessary unless there is a deterioration in clinical condition. This appealing case highlights the importance of raising awareness in recognizing Hamman's syndrome and its significance in distinguishing this condition from Boerhaave's syndrome, as shown in Table 2, which carries a high mortality rate. To our knowledge, this is the first Australian case report to describe Hamman's syndrome in a child with TIDM presenting with DKA.

## 4. Conclusion

This case highlights the importance of recognizing Hamman's syndrome as a potential complication of DKA in pediatric patients. Prompt diagnosis and management, along with differentiation from more severe conditions like Boerhaave's syndrome, are crucial for ensuring favorable outcomes. Further awareness and understanding of this rare syndrome are essential for optimal patient care and management.

## Figures and Tables

**Table 1:** Initial vital signs and venous blood gas parameters.

*Vitals at presentation*	
Heart rate	122 beats/min
Respiratory rate	50 breaths/min
Blood pressure	160/100 mmHg

*Venous blood gas*	
pH	<7.0
Base excess	−20
Bicarbonate	3 mmol/L
Anion gap	32 mmol/L

*Other results*	
Urea	5.2 mmol/L
Creatinine	58 *μ*mol/L
Corrected sodium	139 mmol/L

**Table 2:** Comparison between Hamman's syndrome and Boerhaave's syndrome.

Characteristics	Hamman's syndrome [9]	Boerhaave's syndrome [10]
Definition	Spontaneous mediastinal emphysema without esophageal perforation	Transmural perforation of the esophagus caused by a sudden increase in intraoesophageal pressure
Etiology	Spontaneous, idiopathic	Often associated with forceful vomiting, coughing, or retching
Clinical features	Subcutaneous emphysema, chest pain, dyspnea, and crepitus	Severe chest pain, vomiting, dyspnea, and shock
Diagnostic features	Imaging (e.g., CT and X-ray) may show mediastinal air and subcutaneous emphysema	Imaging (e.g., CT, X-ray, and endoscopy) may show perforation and pneumomediastinum
Management	Conservative management, with close monitoring and supportive care	Urgent surgical intervention (e.g., thoracotomy)
Prognosis	Generally good, with spontaneous resolution within a few days to weeks	Mortality rates up to 40% if untreated or with delayed intervention

## Data Availability

The data used to support the findings of this study are available on request from the corresponding author. Refined data are included within the article.
